# Fixed-point oblivious quantum amplitude-amplification algorithm

**DOI:** 10.1038/s41598-022-15093-x

**Published:** 2022-08-22

**Authors:** Bao Yan, Shijie Wei, Haocong Jiang, Hong Wang, Qianheng Duan, Zhi Ma, Gui-Lu Long

**Affiliations:** 1 State Key Laboratory of Mathematical Engineering and Advanced Computing, Zhengzhou, 450001 China; 2 State Key Laboratory of Low-Dimensional Quantum Physics and Department of Physics, Tsinghua University, Beijing, 100084 China; 3 Beijing Academy of Quantum Information Sciences, Beijing, 100193 China; 4 Institute of Information Technology, Information Engineering University, Zhengzhou, 450001 China; 5 Beijing National Research Center for Information Science and Technology and School of Information, Tsinghua University, Beijing, 100084 China; 6 Frontier Science Center for Quantum Information, Beijing, 100084 China

**Keywords:** Mathematics and computing, Physics

## Abstract

The quantum amplitude amplification algorithms based on Grover’s rotation operator need to perform phase flips for both the initial state and the target state. When the initial state is oblivious, the phase flips will be intractable, and we need to adopt oblivious amplitude amplification algorithm to handle. Without knowing exactly how many target items there are, oblivious amplitude amplification also suffers the “soufflé problem”, in which iterating too little “undercooks” the state and too much “overcooks” the state, both resulting in a mostly non-target final state. In this work, we present a fixed-point oblivious quantum amplitude-amplification (FOQA) algorithm by introducing damping based on methods proposed by A. Mizel. Moreover, we construct the quantum circuit to implement our algorithm under the framework of duality quantum computing. Our algorithm can avoid the “soufflé problem”, meanwhile keep the square speedup of quantum search, serving as a subroutine to improve the performance of quantum algorithms containing oblivious amplitude amplification procedure.

## Introduction

Quantum amplitude amplification algorithms^1–3^ have a vast variety of applications in the field of quantum computing, such as quantum state preparation^4–6^, quantum probability algorithm^7,8^, quantum counting^9–11^ and so on. As a generalization of Grover’s quantum search algorithm^12–15^, quantum amplitude amplification can be described as rotations in the 2-dimensional Hilbert plane extended by the target state $\left| t\right\rangle$ and the original source state $\left| s\right\rangle$. Consider an unsorted database which contains *N* items with *M* target items, the quantum amplitude amplification algorithm can amplify the amplitude of the target states to *O*(1) through $O(\sqrt{N/M})$ Grover iterations. While the classical methods need approximately *O*(*N*/*M*) queries. The necessary condition that the quantum amplitude amplification algorithms based on Grover’s rotation operator work is the ability to make the phase flips for both the target state $\left| t\right\rangle$ and the original state $\left| s\right\rangle$. In some cases, the original state is oblivious, for example, the unsorted database contains both the index register and the content register(the state of which is usually oblivious), then we should use the oblivious amplitude amplification algorithm^16^.

**Figure 1:**
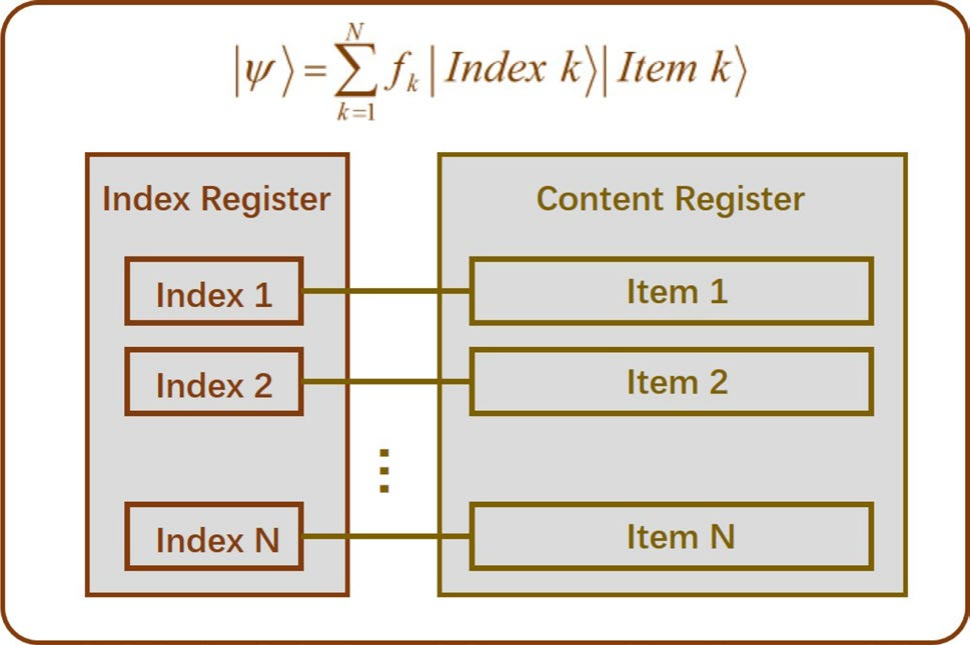
Quantum search model including both the index register and the content register. The index register which stores the index information in superposition state, and the content register stores the content information(usually oblivious) corresponds to index information. The two registers are in entangled states during the process of searching.

The oblivious amplitude amplification method was first introduced by Berry et al. in 2014 and used to deal with the sparse Hamiltonian system simulation problem^16,17^. The method can be used for the unsorted database searching problem which contains both the index register and the content register. As shown in Fig. 1, the two registers are in entangled states during the process of searching. The oblivious amplitude amplification algorithm works by amplifying the amplitude of the index state together with the target state. Consider a unitary operator *U* which can implement another unitary *V* (generally unknown) with some probability. The oblivious amplitude amplification method can implement *V* with high probability through a version of amplitude amplification similar to the original Grover’s search algorithm. Specifically, suppose *U* and *V* are unitary operators on $n+1$ and *n* qubits respectively, and let $\theta \in (0,\pi /2)$. For an arbitrary *n*-qubit state $\left| \varphi \right\rangle$ , we have

1 $$\begin{aligned} U\left| 0\right\rangle \left| \varphi \right\rangle =\sin {\theta }\left| 0\right\rangle V\left| \varphi \right\rangle +\cos {\theta \left| 1\right\rangle \left| \phi \right\rangle }, \end{aligned}$$

where $\left| \phi \right\rangle$ is an *n*-qubit state depends on $\left| \varphi \right\rangle$. Let $R:=(\left| 0\right\rangle \left\langle 0\right| -\left| 1\right\rangle \left\langle 1\right| )\otimes I$, $S:=-URU^{\dagger }R$, then for any $k\in \mathbf {N}$,

2 $$\begin{aligned} S^kU\left| 0\right\rangle \left| \varphi \right\rangle =\sin (2k+1)\theta \left| 0\right\rangle V\left| \varphi \right\rangle +\cos (2k+1)\theta \left| 1\right\rangle \left| \phi \right\rangle . \end{aligned}$$

Here we notice that *R* is a Pauli *Z* operator acting on the index register instead of the reflection about the original state, which makes oblivious amplitude amplification algorithm enjoy different application scenarios.

Oblivious amplitude amplification method suffers the same “soufflé problem”^18,19^ with those based on Grover’s rotation operator. When the exact number of target items in the database is unknown, there is no knowing when to stop the iteration properly. Iterating too little “undercooks” the state and too much “overcooks” the state, both leaving a mostly non-target final state. In 2005, Grover presented the fixed-point quantum search algorithm to tackle this problem^20^. The rotation phase of the original Grover operator is modified to $\pi /3$. As a result, the amplitude of the target state increases monotonically as the number of iterations grows. However, the amplification efficiency is sacrificed. For the $O(2^{n/2})$ original Grover iterations, the fixed-point version needs $O(3^n)$ iterations. Other fixed-point search algorithms have also been proposed subsequently and the quantum square speedup efficiency regained^19,21–26^. In 2009, Mizel^22^ adopted the damping of dissipative system to deal with the “soufflé problem” and the overhead is only 1.5 times that of the original Grover, namely $O(1.5\sqrt{N/M})$. In 2017, Dalzell et al. realized the fixed-point version of the Grover algorithm under adiabatic model^24^, meanwhile mentioned the idea of applying fixed-point search to oblivious amplitude amplification. In 2019, Lei et al. implemented Mizel’s damping version of fixed-point search based on duality quantum computation model^25^.

In this paper, we present the fixed-point oblivious quantum amplitude-amplification (FOQA) algorithm based on the damping method. We have constructed the explicit quantum circuit to implement the algorithm in the framework of duality quantum computation. The core operator for iteration is implemented by the linear combination of unitaries (LCU) which is one of the major methods for quantum algorithm design. The new algorithm can avoid the “soufflé problem” while maintaining the quantum square speedup. For an unsorted database with *N* items and *M* target items, the FOQA algorithm using approximately $O(1.5\sqrt{N/M})$ iterations. The rest of this paper is organized as follows. In “The framework of duality quantum computation”, we introduce the main idea and framework of duality quantum computation (or LCU); in “Mizel’s fixed-point quantum search”, an introduction of the Mizel’s fixed-point search method based on damping; We present the FOQA algorithm and the quantum circuit implementation in “FOQA algorithm and its circuit implementation”; Finally, we give the conclusion in “Conclusion”.

## The framework of duality quantum computation

The quantum operations performed by a general quantum computer are unitary transformations^27^, while a duality quantum computer can implement a class of more generalized operators^28^, namely the linear combination of unitaries (LCU), which are usually non-unitary. Inspired by the wave-particle duality of microscopic particles, Long proposed the duality quantum computing model based on the double-slit interference phenomenon in 2006^28–33^. The core functions of this computing model are accomplished by two kinds of generalized computing gates, namely quantum wave division (QWD) and quantum wave combination (QWC), which are two unitaries working on the ancillary qubits. Consider a nonunitary operator $$H=\frac{1}{C}\begin{matrix} \sum _{l=0}^{L-1} \beta _lU_l \end{matrix}$$, here $$C=\sqrt{\begin{matrix} \sum _{l=0}^{L-1} \beta _l^2 \end{matrix}}$$ is the normalization coefficient. This operator, the linear combination of unitaries can be realized in four main steps:

i. Wave division. Suppose the work register is initialized to $\left| \psi _0\right\rangle$. The ancillary qubits are prepared to the initial state $\left| \psi _i\right\rangle$ by the QWD operation,

3 $$\begin{aligned} \left| \psi _i\right\rangle =\frac{1}{C} \sum _{l=0}^{L-1} \beta _l\left| l\right\rangle , \end{aligned}$$

The number of qubits needed in ancillary register is $m=\lceil {\log _2L}\rceil$, the symbol $\lceil {a}\rceil$ means round up to an integer not less than *a*. Here the state of the whole quantum register is:

4 $$\begin{aligned} \left| \Phi _1\right\rangle =\left| \psi _i\right\rangle \left| \psi _0\right\rangle =\frac{1}{C} \sum _{l=0}^{L-1} \beta _l\left| l\right\rangle \left| \psi _0\right\rangle . \end{aligned}$$

ii. Entanglement generation. In this step, a series of ancillary system controlled operators $$\begin{matrix} \sum _{l=0}^{L-1} \left| l\right\rangle \left\langle l\right| \otimes {U_l} \end{matrix}$$ are implemented on the work register. Then the ancillary register and the work register are entangled. The state of the system is transformed into

5 $$\begin{aligned} \left| \Phi _2\right\rangle =\frac{1}{C} \sum _{l=0}^{L-1} \beta _l\left| l\right\rangle U_l\left| \psi _0\right\rangle . \end{aligned}$$

iii. Wave combination. The QWC operation is implemented to integrate the quantum states in *m* qubits of the ancillary register. And the *L* wavelets in the subspace are integrated into the initial state $\left| 0\right\rangle ^{\otimes {m}}$ of the ancillary register,

6 $$\begin{aligned} \left| \Phi _3\right\rangle =\frac{1}{C\sqrt{2^m}}\left| 0\right\rangle ^{\otimes {m}} \sum _{l=0}^{L-1} \beta _lU_l\left| \psi _0\right\rangle . \end{aligned}$$

iv. Post-processing and measurement. If the ancillary register is measured directly, then the state $\left| 0\right\rangle ^{\otimes {m}}$ can be measured with the probability of $p_m$,

7 $$\begin{aligned} p_m=\left\| \frac{1}{C\sqrt{2^m}}\sum _{l=0}^{L-1} \beta _lU_l\left| \psi _0\right\rangle \right\| ^2=\frac{\left\| H\left| \psi _0\right\rangle \right\| ^2}{L}. \end{aligned}$$

Notice that the whole LCU process is successful only when the ancillary register is in state $\left| 0\right\rangle ^{\otimes {m}}$. When *m* is big, the probability $p_m$ can be very small, then the process needs post processing before measurement. The oblivious amplitude amplification method can be used to enlarge the amplitude of the state $\left| 0\right\rangle ^{\otimes {m}}$. It is easy to observe that a general quantum operator $$H=\frac{1}{C}\begin{matrix} \sum _{l=0}^{L-1} \beta _lU_l \end{matrix}$$ can be implemented in the framework of duality quantum computation. Since this operator is usually not unitary, duality quantum computation or the linear combination of unitaries (LCU) can be adopted to broader applications of quantum computing. More details about duality quantum computation or LCU can be found in^28–33^.

## Mizel’s fixed-point quantum search

In 2009, Mizel observed that the transformation between quantum search and classical search can be realized by adjusting the magnitude of damping^22^. When the magnitude of the introduced damping is large enough, the algorithm becomes a pure classical search without any quantum speedup. While the damping is sufficiently small, the search process becomes the quantum Grover algorithm. There is a critical damping value that enables a fixed-point quantum search, and the number of Oracle calls becomes only 1.5 times that of the original Grover. Consider an unsorted database of *N* items, *M* of which are the target items. Here the value *M*/*N* is unknown. The main tool of the algorithm is an Oracle that can recognize the target state by flipping the phase of the target state while leaving the phases of the other quantum states unchanged. First, initialize the register into the equal superposition state

8 $$\begin{aligned} \left| \psi \right\rangle =\frac{1}{\sqrt{N}}\sum _{i=0}^{N-1} \left| i\right\rangle =\cos (\xi /2)\left| \gamma \right\rangle +\sin (\xi /2)\left| \beta \right\rangle . \end{aligned}$$

Here $\sin (\xi /2)=\sqrt{M/N}$, we define $\left| \beta \right\rangle$ the equal superposition of the target statesand $\left| \gamma \right\rangle$ is the equal superposition of $N-M$ non-target states. Grover search process can be described as a series of rotations in the 2-dimensional Hilbert plane (or space) spanned by $\left| \gamma \right\rangle$ and $\left| \beta \right\rangle$. In this space, the Pauli operators can be defined as $\bar{X}=\left| \gamma \right\rangle \left\langle \beta \right| +\left| \beta \right\rangle \left\langle \gamma \right|$, $\bar{Y}=i\left| \beta \right\rangle \left\langle \gamma \right| -i\left| \gamma \right\rangle \left\langle \beta \right|$, $\bar{Z}=\left| \gamma \right\rangle \left\langle \gamma \right| -\left| \beta \right\rangle \left\langle \beta \right|$. Here $\bar{Z}$ operator is the Oracle of the Grover algorithm used to rotate the phases of the target states. Another important operator in Grover algorithm is the so called “inversion about the mean”, which can be constructed as $\bar{E}=2\left| \psi \right\rangle \left\langle \psi \right| -I$. Then the whole Grover rotation operator can be constructed as: $G=\bar{E}\bar{Z}=\exp (-i\xi \bar{Y})$. After *k* Grover iterations, the system becomes

9 $$\begin{aligned} G^k\left| \psi \right\rangle =\sin ((2k+1)\xi /2)\left| \gamma \right\rangle +\cos ((2k+1)\xi /2)\left| \beta \right\rangle . \end{aligned}$$

Mizel introduced an ancillary qubit to indicate the proportion of the target states. Assume the ancillary qubit is initialized into $\left| 1\right\rangle$, Mizel’s fixed-point search algorithm can be described as follows.

Step1. If the state of work register is target state, then rotate the ancillary qubit by $e^{-i\alpha {Y}}$. Here the phase angle $\alpha$ is used to control the magnitude of damping. The value of damping will change with the iteration. More details about the changing rule of $\alpha$ could be found in^22^. This step needs to call the Oracle $\bar{Z}$ and it can be expressed as$[e^{-i\alpha Y}\frac{I-\bar{Z}}{2}+\frac{I+\bar{Z}}{2}]$.

Step2. If the ancillary qubit is in its original state $\left| 1\right\rangle$, then apply the Grover rotation to the work qubits. This step can be described as: $[\frac{I-Z}{2}G+\frac{I+Z}{2}]$.

Step3. Measure the ancillary qubit. If it is in the state $\left| 1\right\rangle$, return to the first step and go on to the next iteration. Else, return the target state.

Here *Y*, *Z* are single qubit Pauli operators, namely *Y* gate and *Z* gate.

## FOQA algorithm and its circuit implementation

**Figure 2:**
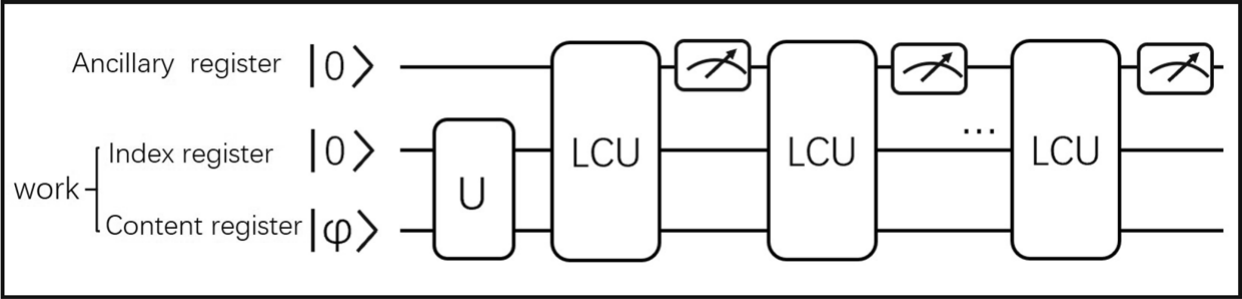
Quantum circuit for fixed-point oblivious quantum amplitude-amplification. It is an iteration circuit based on the LCU operator. After each iteration, measurement is performed on the ancillary qubit. If the result is $\left| 0\right\rangle$, move to next the iteration. Else, return the target state.

**Figure 3:**
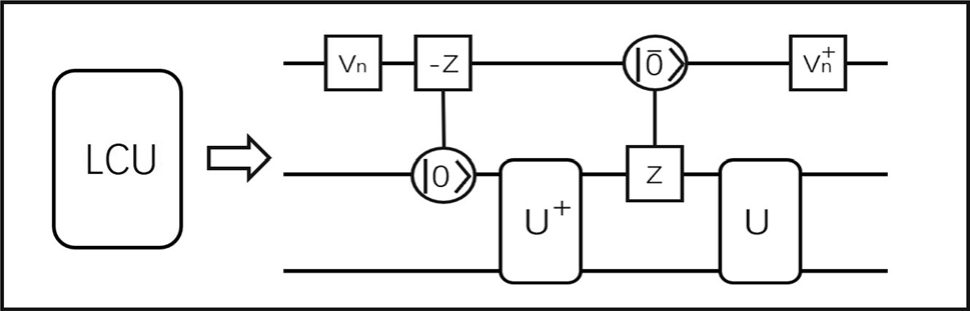
The quantum circuit for implementing the LCU operator in the framework of duality quantum computation. At the begining and end of the circuit, the wave division unitary $V_n$ and the wave combination unitary $V_n^{\dagger }$ are performed. The function of the control-operators is to generate the entanglement.

In order to realize fixed-point oblivious quantum amplitude-amplification (FOQA) algorithm via Mizel’s damping method, we refer to the transformation technique in^25^ and implement the algorithm using linear combination of unitaries (LCU). As shown in Fig. 2, the amplification process requires three quantum registers, namely the single qubit ancillary register, the index register and the content register. The latter two registers are collectively referred to as the working register. In the application, the unsorted database state to be retrieved needs to be prepared by the unitary transformation *U* applied to the working register. This operation makes the index states entangle with the content states. Then, FOQA can be constructed by using iterations of the LCU circuit (see Fig. 3 for details).

After each iteration, the ancillary register needs to be measured. If the result is $\left| 0\right\rangle$, the process will be completed and we’ll get the target state in work register. Otherwise, proceed to the next iteration. The process of the whole algorithm is as follows:

I. Initialize the circuit. Prepare the three registers into state $\left| 0\right\rangle \left| 0\right\rangle \left| \varphi \right\rangle$.
II. Generate the database state by the *U* operator performing on work register. We have $U\left| 0\right\rangle \left| \varphi \right\rangle =\sin (\theta )\left| 0\right\rangle V\left| \varphi \right\rangle +\cos (\theta )\left| 1\right\rangle \left| \phi \right\rangle ,$ here $V\left| \varphi \right\rangle$ is the target state for searching and the corresponding index state is $\left| 0\right\rangle$.
III. Utilize the LCU circuit to amplify the amplitude of the target state. The LCU circuit will change the phases of index states together with content states.
IV. Measure the ancillary register. If it is $\left| 0\right\rangle$, return to step III and go on to the next iteration. Else, return the target state $V\left| \varphi \right\rangle$.

The above is the workflow of FOQA algorithm. The following is to design the quantum circuit based on linear combination of unitary operators. We learn from the methods of Lei et al.^22,25^. According to “Mizel’s fixed-point quantum search”, the quantum operation corresponding to Mizel’s damping fixed-point search can be constructed as follows:

10 $$\begin{aligned} \begin{aligned} \bar{U}=\,&[\left| 0\right\rangle \left\langle 0\right| \otimes {S}+\left| 1\right\rangle \left\langle 1\right| \otimes {I_2}\otimes {I_3}][e^{-i\alpha _n Y}\otimes \left| 0\right\rangle \left\langle 0\right| \otimes {I_3}+I_1\otimes \left| 1\right\rangle \left\langle 1\right| \otimes {I_3}]\\ =\,&[\left| 0\right\rangle \left\langle 0\right| \otimes {UZU^{\dagger }}+\left| 1\right\rangle \left\langle 1\right| \otimes {I_2}\otimes {I_3}][-V_n^{\dagger }ZV_n\otimes \left| 0\right\rangle \left\langle 0\right| \otimes {I_3}+I_1\otimes \left| 1\right\rangle \left\langle 1\right| \otimes {I_3}]. \end{aligned} \end{aligned}$$

Here $I_i$ represents for the identity of the $i_{th}$($i=1,2,3$) register and the subscript *n* represents the *n*-th iteration. We have used the equation $e^{-i\alpha _n{Y}}=ZV_n^{\dagger }ZV_n$ in the transformation of (10), here

11 $$\begin{aligned} V_n=e^{-i\alpha _n Y/2}=\begin{pmatrix} \cos {\frac{\alpha _n}{2}}&{} -\sin {\frac{\alpha _n}{2}}\\ \sin {\frac{\alpha _n}{2}}&{} \cos {\frac{\alpha _n}{2}} \end{pmatrix}, \end{aligned}$$

Further more, utilize the identites $V_nV_n^{\dagger }=I_1, UU^{\dagger }=I_2\otimes {I_3}$, the LCU operator can be further simplified as:

12 $$ \begin{aligned} \bar{U}=&V_n^{\dagger }\otimes U(\left| \bar{0}\right\rangle \left\langle \bar{0}\right| \otimes {Z}\otimes {I_3}+\left| \bar{1}\right\rangle \left\langle \bar{1}\right| \otimes {I_2}\otimes {I_3})I_1\otimes U^{\dagger }(-Z\otimes \left| 0\right\rangle \left\langle 0\right| \otimes {I_3}+I_1\otimes \left| 1\right\rangle \left\langle 1\right| \otimes I_3)V_n, \end{aligned} $$

here

13 $$ \begin{aligned}   \left| {\bar{0}} \right\rangle  =  & V_{n} \left| 0 \right\rangle  = \cos \frac{{\alpha _{n} }}{2}\left| 0 \right\rangle  + \sin \frac{{\alpha _{n} }}{2}\left| 1 \right\rangle , \\    \left| {\bar{1}} \right\rangle  =  & V_{n} \left| 1 \right\rangle  =  - \sin \frac{{\alpha _{n} }}{2}\left| 0 \right\rangle  + \cos \frac{{\alpha _{n} }}{2}\left| 1 \right\rangle .{\text{ }} \\  \end{aligned}  $$

The quantum circuit implementation of this operator is shown in Fig. 3.

After *n*-th iteration, measure the ancillary qubit. If we get $\left| 0\right\rangle$, then move forward to the $(n+1)$-th iteration. At this time, suppose the work register stay in the state:

14 $$\begin{aligned} \left| \Psi _0\right\rangle =\left| 0\right\rangle (t_n\left| 0\right\rangle V\left| \varphi \right\rangle +s_n\left| 1\right\rangle \left| \phi \right\rangle ). \end{aligned}$$

Here $t_n$ represents for the amplitude of the target state $\left| 0\right\rangle V\left| \varphi \right\rangle$ after *n*-th iteration, while $s_n$ represents for the amplitude of the non-target state $\left| 1\right\rangle \left| \phi \right\rangle$.

Step 1. Wave division. Perform the operator $V_n$ to ancillary qubit, the whole wave function becomes:

15 $$\begin{aligned} \left| \Psi _1\right\rangle =(\cos \frac{\alpha _n}{2}\left| 0\right\rangle +\sin \frac{\alpha _n}{2}\left| 1\right\rangle )(t_n\left| 0\right\rangle V\left| \varphi \right\rangle +s_n\left| 1\right\rangle \left| \phi \right\rangle ). \end{aligned}$$

Step 2. Entanglement generation. If the index qubit is in $\left| 0\right\rangle$, apply the -*Z* operator to the ancillary register. The equivalent unitary transformation of this step is: $(-Z\otimes {\left| 0\right\rangle \left\langle 0\right| }\otimes {I_3}+I_1\otimes {\left| 1\right\rangle \left\langle 1\right| }\otimes {I_3})$ . The state of the whole system changes to

16 $$\begin{aligned} \begin{aligned} \left| \Psi _2\right\rangle =&t_n\left( -\cos \frac{\alpha _n}{2}\left| 0\right\rangle +\sin \frac{\alpha _n}{2}\left| 1\right\rangle \right) \left| 0\right\rangle V\left| \varphi \right\rangle +s_n\left( \cos \frac{\alpha _n}{2}\left| 0\right\rangle +\sin \frac{\alpha _n}{2}\left| 1\right\rangle \right) \left| 1\right\rangle \left| \phi \right\rangle . \end{aligned} \end{aligned}$$

Step 3. Perform the $U^{\dagger }$ operator to the work register. We have

17 $$\begin{aligned} \begin{aligned} \left| \Psi _3\right\rangle&=t_n\left( -\cos \frac{\alpha _n}{2}\left| 0\right\rangle +\sin \frac{\alpha _n}{2}\left| 1\right\rangle \right) \left( \sin \theta \left| 0\right\rangle \left| \varphi \right\rangle +\cos \theta \left| 1\right\rangle \left| \chi \right\rangle \right) \\&\quad +s_n\left( \cos \frac{\alpha _n}{2}\left| 0\right\rangle +\sin \frac{\alpha _n}{2}\left| 1\right\rangle \right) \left( \cos \theta \left| 0\right\rangle \left| \varphi \right\rangle -\sin \theta \left| 1\right\rangle \left| \chi \right\rangle \right) . \end{aligned} \end{aligned}$$

Here we have used the results in^16^ that

18 $$ \begin{aligned}   U^{\dag } \left| 0 \right\rangle V\left| \varphi  \right\rangle  =  & \sin \theta \left| 0 \right\rangle \left| \varphi  \right\rangle  + \cos \theta \left| 1 \right\rangle \left| \chi  \right\rangle , \\    U^{\dag } \left| 1 \right\rangle \left| \phi  \right\rangle  =  & \cos \theta \left| 0 \right\rangle \left| \varphi  \right\rangle  - \sin \theta \left| 1 \right\rangle \left| \chi  \right\rangle . \\  \end{aligned}  $$

Step 4. Entanglement generation. If the ancillary qubit is in $\left| \bar{0}\right\rangle$, perform the *Z* operator to the ancillary register. The equivalent unitary transformation of this step is: $(\left| \bar{0}\right\rangle \left\langle \bar{0}\right| \otimes {Z}\otimes {I_3}+\left| \bar{1}\right\rangle \left\langle \bar{1}\right| \otimes {I_2}\otimes {I_3})$, and the whole wave function becomes

19 $$\begin{aligned} \begin{aligned} \left| \Psi _4\right\rangle =&-t_n\cos \alpha _n\left| \bar{0}\right\rangle (\sin \theta \left| 0\right\rangle \left| \varphi \right\rangle -\cos \theta \left| 1\right\rangle \left| \chi \right\rangle )+s_n\left| \bar{0}\right\rangle (\cos \theta \left| 0\right\rangle \left| \varphi \right\rangle +\sin \theta \left| 1\right\rangle \left| \chi \right\rangle )\\ {}&+t_n\sin \alpha _n\left| \bar{1}\right\rangle (\sin \theta \left| 0\right\rangle \left| \varphi \right\rangle +\cos \theta \left| 1\right\rangle \left| \chi \right\rangle ). \end{aligned} \end{aligned}$$

Step 5. Wave combination. Perform the $V_n^{\dagger }$ operator on the ancillary registerand implement *U* operator on the work register. We have

20 $$\begin{aligned} \begin{aligned} \left| \Psi _5\right\rangle =\sqrt{p_{n+1}}\left| 1\right\rangle \left| 0\right\rangle V\left| \varphi \right\rangle +\sqrt{1-p_{n+1}}\left| 0\right\rangle (t_{n+1}\left| 0\right\rangle V\left| \varphi \right\rangle +s_{n+1}\left| 1\right\rangle \left| \phi \right\rangle ), \end{aligned} \end{aligned}$$

where we have

21 $$ \begin{aligned}   p_{{n + 1}}  =  & \sin ^{2} \alpha _{n} |t_{n} |^{2} , \\    t_{{n + 1}}  =  & \frac{{t_{n} \cos \alpha _{n} \cos 2\theta  + s_{n} \sin 2\theta }}{{\sqrt {1 - p_{{n + 1}} } }}, \\    s_{{n + 1}}  =  & \frac{{ - t_{n} \cos \alpha _{n} \sin 2\theta  + s_{n} \cos 2\theta }}{{\sqrt {1 - p_{{n + 1}} } }}. \\  \end{aligned}  $$

At this time, the ancillary register is measured and the state $\left| 1\right\rangle$ will be obtained with the probability of $p_{n+1}$. According to Eq. (4), the quantum state of the work register is $\left| 0\right\rangle V\left| \varphi \right\rangle$, which means the amplifying process is successful. Otherwise, proceed to the next iteration until the state $\left| 1\right\rangle$ is obtained. The above is the process of FOQA algorithm implemented by the LCU quantum circuits. The failure probability of the algorithm after *n* iterations is equivalent to the probability of getting $\left| 0\right\rangle$ everytime when measuring the ancillary register. Thus, the failure rate of *n* iterations is: $$q_n=\begin{matrix} \prod _{k=1}^n (1-p_k) \end{matrix}$$. When the damping values $\alpha _n$ are selected according to the Ref.^25^, the theoretical result of the success rate of the algorithm in this paper (see Eq. ()) is the same as that in Ref.^22^. According to the analysis in the literatures^22,25^, with the iteration number increasing, the failure probability will approach to zero. The average number of iterations required before we get $\left| 1\right\rangle$ on ancillary qubit is $O(1.5\sqrt{N/M})$.

## Conclusion

Quantum amplitude amplification methods based on Grover’s operator suffer the “soufflé problem” when the exact number of target items is unknown. In that case, iterating too little “undercooks” the state and too much “overcooks” the state, both leaving a mostly non-target final state. The oblivious amplitude amplification is an alternative quantum method similar to Grover’s, and can be used in different application scenarios like searching unsorted database which contains both index register and content register. However, it faces the same “soufflé problem” as Grover. In this work, we give the fixed-point oblivious quantum amplitude-amplification (FOQA) algorithm based on the damping method introduced by Mizel. We also present the quantum circuit to implement the algorithm adopting the idea of duality quantum computation. The core operator for iteration is implemented by the LCU method. Our new algorithm can avoid the “soufflé problem” while maintaining the quantum-square speedup. In the search case when the proportion of the target states is unknown, the target unitary *V* can be implemented with the success rate *O*(1) by our new algorithm in approximately $O(1.5\sqrt{N/M})$ iterations, which is as efficient as the fixed-point algorithm of Mizel. In the design of the duality quantum circuits, we adopt a similar transformation technique to that in Ref.^25^, and achieves the same theoretical results.

FOQA algorithm can be used as a subroutine in any scenario where oblivious amplitude amplification is applied^34,35^. The research of gate-model quantum computer has achieved great development in recent years^36–45^. Quantum superemacy or quantum advantage has been fulfilled successively in different quantum systems^46–48^. In the era of noisy intermediate scale quantum (NISQ)^49^, variational quantum algorithms (VQAs) provide a general framework which can be used to solve a wide variaty of problems^50–54^. The FOQA algorithm can be combined with VQAs to improve the fidelity of the final solution in the future.
